# Letter from L. G. Hardman, M. D., Guy’s Hospital, London, Eng.

**Published:** 1890-07

**Authors:** L. G. Hardman

**Affiliations:** Guy’s Hospital, London, Eng.


GUY'S HOSPITAL, LONDON, ENG., June, '90.

MR. EDITOR—It is perhaps very often your readers are burdened, or at any rate the space in your journal is occupied, by letters from doctors who are visiting New York, London, Paris, Berlin or some great medical center, in which the doctor (the writer) is much more interested than the subscriber to your most valuable journal. However, I will venture a short letter, hoping I may at least present some facts which are not known to some of your readers who are not able to keep abreast of the profession. First, I will mention surpra-pubic cystotomy which is being done quite frequently now in London for stone, enlarged prostate and tubercular disease of the mucous membrane of the organ, as well as for other conditions of the mucous membrane in which all other treatments have failed. In the operation for stone, the patient recovers completely and the risk is not so great in this operation now since antiseptics are used, as it was believed to be at one time. For the prostatic enlargement I refer to the hypertrophy of the so-called middle lobe more especially.

In the two cases of Mr. Hill at the University Hospital, the recovery was complete after the removal of this portion of the prostate, and in one of the cases thirteen stones were removed at the same time.

I will note next the early treatment of the enlarged prostate as brought out by Mr. Reginald Harrison, which I do not believe is as generally practiced in our section as it should be, namely, the bougie. The three kinds, which he uses and recommends, are firstly, the belly bougie as he calls it; secondly, the Olive point, and thirdly, the whip, which is an instrument of his own; however, he prefers the first instrument.

The object of this treatment is, if commenced early and used two or three times a week, by careful and gentle introduction of the instrument, pushing the enlarged bodies away from the urethral opening, and as it were producing a groove for the escape of urine and thereby preventing the large accumulation of urine which results from the obstruction and necessitates the use of the catheter. When the use of the catheter is once commenced and kept up, which is very often the case, the walls of the bladder atrophy, and so the power which it had to expel the urine and overcome the obstruction is lessened, and the catheter is continued the remainder of life. He claims that almost every man, who arrives at the age of sixty, has an enlarged prostate, quite large enough in size to obstruct the flow of urine, if it was not for nature pushing away and making a groove, as it were, for the flow of the urine, and thus the would-be obstruction is overcome in a great many cases.

Now by the method here suggested the same thing is accomplished. The early use of the bougie (not catheter) channels out a road, and great and permanent good is done. He thinks sometimes, where the bladder is full of urine in old prostatic troubles, and you are compelled to use the catheter, that it is not best in every case to draw off all the urine at one time. He considers ergot the best bladder tonic.

Antiseptics are used throughout London in all of the hospitals. In some of them, namely Guy's, the carbolic spray as used by Sir Joseph Lister, is now used in every operation. Mr. N. Thornton, at the Samaritan, also uses it in all of his abdominal sections, and by the way, he is considered one of the best operators in that line in London. Sir Spencer Wells seldom uses it, neither does Mr. Thomas Kieth. They all, however, use antiseptics and antiseptic dressings. The ones most in use are the bichloride of mercury, carbolic acid, cyanide of mercury, iodoform and permanganate of potash. Sir Spencer Wells uses five per cent. solution of carbolic acid; however, he does not use it in the abdominal cavity, and by the way, I have it from him that he does not wash out the cavity after his laparotomies, as is the modern fashion, neither does he put in a drainage tube; has not used one for twelve years. His manner of closing the abdominal wound is the simplest I have seen. He uses a double needle suture of silk, passing one from each side under the peritoneal surface, and coming out through the skin, and including all of the tissues which have been cut through, and bringing it together in this way, using as many sutures as the wound requires. He is certainly a remarkable man; he is now seventy-two years of age, and is doing constant work.

Thos. Kieth uses the drainage tube in about one in ten cases. I had the pleasure of seeing Mr. Tait's operation for lacerated perineum, at Soho Hospital for women. It is certainly a much simpler operation than almost any done on the perineum. I will not go into the details of the operation, however.

In almost all of the eye operations they use cocaine, four per cent. solution, in cataract, irridectomy, etc. They use the cocaine almost exclusively in adult subjects.

It is wonderful to see the number of hernial operations now being done in all of the hospitals for radical cure, and with fair success. The needles and ligatures which are used now are the flat, curved and straight needle, and silk worm gut.

I wish to call attention also to the splint now used almost exclusively in Guy's Hospital for fractures of the femur: namely, Hodgens' Splint. This splint is really a modification of Hodgens, which they use, but is called by his name. He is a St. Louis surgeon. It certainly renders the patient very comfortable; at any rate, the patients say so. The surgeons claim as good or better results from this, than any other splint, besides the great comfort to the patients.

The splint now used a great deal at Guy's Hospital for fractures of the tibia and fibula is known as Mr. Croft's splint, which is about the same in substance as the Bavarian splint. In Mr. Croft's the flannel is saturated with the plaster of Paris and covered by starch bandages until it dries, and then the bandage is cut on top of the leg, and the splint or plaster on each side can be removed very easily and nicely. There are a large number of operations of all kinds done at Guy's Hospital; there are from five to fifteen daily.

The Lying-in Charity at Guy's is by far the largest in London.

The number of cases last year was 2,924. There are many other things here of great interest to me, but I will close, and perhaps write you again. L. G. HARDMAN.

